# Recent advances in forensic anthropology

**DOI:** 10.1080/20961790.2018.1466384

**Published:** 2018-06-04

**Authors:** Douglas H. Ubelaker

**Affiliations:** Department of Anthropology, National Museum of Natural History, Smithsonian Institution, Washington, DC,United StatesUbelaked@Si.Edu

Forensic anthropology involves diverse applications of anthropological knowledge to medico-legal problems. While the applications are evidence-driven, the available scientific methodology and foundation have developed through decades of research and experience. The roots of this field are anchored in comparative human anatomy but methodology has developed through experimentation, the assemblage of documented collections and databases and thoughtful research design. While forensic anthropology represents a mature scientific field, it continues to evolve and advance through new, innovative global research. Much of this progress is fuelled by issues encountered in casework. The unique evidence and problems presented in forensic cases call for the very best scientific approaches available. Usually, the correct approaches and solutions can be found in the existing scientific literature. However, sometimes the unique issues presented by the casework cannot be addressed adequately with the existing techniques. These situations stimulate forensic anthropologists to seek new solutions through targeted research.

This Special Issue presents research advances in several areas of forensic anthropology that have sustained rapid, recent progress. While our journals continually reveal new information in all aspects of forensic anthropology, several areas of investigation have registered particularly strong academic interest featuring innovative research.

## Molecular analysis of skeletal evidence

Successful recovery and analysis of DNA has dramatically affected many areas of forensic science. In the field of forensic anthropology, molecular analysis can yield highly accurate information regarding the sex of the individual represented and provide positive identification [1]. Molecular approaches also can contribute to ancestry evaluation and species recognition. The use of DNA for positive identification has had a major impact on the practice of forensic anthropology and related fields of forensic science.

While the merits and contributions of DNA analysis are profound, many related issues express the need for new, innovative research and technological development. Frequently, evidence submitted for forensic anthropological analysis is not in pristine condition. In many cases, recovered remains are incomplete and/or extremely degraded due to criminal activity and/or taphonomic factors. Some site investigations produce only small fragments where even species is not apparent. Decisions need to be made regarding what areas of bone or tooth should be examined. Since DNA analysis is an expensive and destructive process, these decisions are critical and can affect the outcome of the case. Of course, decisions regarding the type of DNA analysis also are critical and largely driven by both the availability of the antemortem information and the nature of the evidence. Experimentation and casework experience have greatly improved approaches to these issues.

## Migrant identification

Deaths related to the global movement of undocumented people across national borders present major forensic challenges. Even within countries, identification of citizens can be difficult with incomplete evidence and lack of information regarding missing persons. These problems are greatly exacerbated when different countries are involved and the international movement of the person represented is not registered officially. Such cases call for extraordinary investigation, thoughtful forensic analysis and international communication. These efforts can strain the available local resources and often fall short of positive identification.

Recent years have witnessed remarkable efforts to address the identification of deceased, undocumented border crossers. These initiatives have involved international cooperation, careful exhumation procedures, comprehensive anthropological analysis and new techniques such as isotope analysis to identify the likely regions/countries of origin.

## Search, detection and recovery

The entire process of forensic anthropological investigation begins with the procedures of search, detection and recovery. Improper or inadequate detection and recovery of human remains can compromise the downstream analysis and interpretation. While the traditional techniques of surface survey and excavation continue to be needed, new approaches, especially those using advanced technology offer significant advances.

Search procedures can be especially challenging when only very general information is available regarding the likely location of human remains. Topographic features can present limitations, especially with dense vegetation and other ground cover. Investigations of humanitarian and human rights issues can present special search and recovery challenges when information suggests that wells, cisterns, sewer systems, mass graves or disposal in water were involved. Confronted with these problems, researchers have devised innovative new approaches to improve the probability of success.

## Commingling analysis

Secondary deposits of human remains or those that have sustained significant disturbance involve loss of normal bone articulation patterns. When multiple individuals are involved, the resulting commingling presents challenges to determine the number of persons represented and to assemble remains of individuals for analysis, identification and return to families. Traditional approaches to commingling problems have involved sorting by the type and side (left or right) of bone, age at death, bone size and maturation, sex and pathological conditions. In some skeletal assemblages, taphonomic indicators can be helpful as well.

Once obvious sorting has been completed, questions persist regarding bone morphology related to individuals. Could a robust femur relate to a robust humerus and represent one individual? Recent advances in commingling analysis address this issue. New databases and computerized techniques establish the probabilities that different bones could relate to the same individual. Applications refine the determination of the number of individuals represented and facilitate analysis aimed at identification.

## Biomechanics of bone trauma

A primary function of anthropological analysis relates to the interpretation of bone trauma. Anthropologists must differentiate the skeletal alterations representing perimortem trauma from those relating to antemortem injury, developmental features or postmortem and taphonomic factors. Assessment of the biomechanical factors involved plays a key role in any interpretation. Knowledge of biomechanical principles is required to explain fracture patterns and other alterations likely related to perimortem trauma. Interpretation of bone trauma can be challenging. Such challenges have led to greater understanding of the principles involved and experimental work designed to improve interpretation.

## Decomposition research

Major new initiatives in forensic anthropology have focused on decomposition research. Experiments involving both humans and non-human animals have revealed great detail about the process and variation of soft tissue decomposition and hard tissue alteration. In general, such research has elucidated the many factors that influence both the nature and timing of the decomposition process. Clearly temperature and location (surface, in-ground, aquatic, etc.) have long been regarded as key factors. Research has also indicated that soil conditions, moisture, body composition, body condition, presence of clothing or enclosures, funerary treatment and many other factors can influence the process. Such information is needed to properly assess time since death (post-mortem interval) and post-mortem events related to criminal activity.

## Bone microscopy

In 1965, Ellis R. Kerley [2] published a technique that allowed age at death to be estimated from microscopic examination of features in human compact bone from the femur, tibia and fibula. Kerley's procedure involved the examination of primary osteons, secondary osteons, osteon fragments and the extent of remaining circumferential lamellar bone. This approach gained recognition due to its reported accuracy and the fundamental processes of bone formation and remodelling that it expressed. Since 1965, the technique has undergone many revisions and expansions for application to other bones of the skeleton. Research also has revealed how bone microscopic examination can provide useful information on many issues of forensic anthropological analysis.

## Isotope analysis

For decades, analysis of elemental stable isotopes has offered key anthropological information related to diet. Stable carbon isotopes recovered from human tissues have revealed if diet focused on plants with a C_3_ photosynthetic pathway or a C_4_ pathway and the herbivores that fed upon them. Analysis of nitrogen isotopes provides insight into the trophic level of human diet. In anthropological studies of ancient populations, such information is crucial to interpretations of dietary and horticultural practices.

Recently, researchers have applied the concepts of isotopic analysis to examine the geographical origin of human remains. When unidentified human remains are recovered in forensic contexts, investigators question if they represent someone who lived in the area of recovery or from somewhere else. This question is especially relevant in cases involving terrorism and unidentified possible migrants. Using a battery of stable isotope analyses, researchers can determine if the isotopic signatures from the unknown match local baseline data. If not, attempts can be made to determine from what geographic area the unknown originated. This exciting new area of forensic science investigation depends on the assemblage of baseline data from appropriate geographic regions.

## Facial imaging

Forensic anthropologists relate to issues of facial imaging in facial approximation, craniofacial photographic superimposition and interpretations of surveillance images. Facial approximation refers to the process of estimating the living facial image of a person from the evidence presented by a recovered skull. This technique is used to reach out to the public for leads in missing persons that could culminate in identification using other methods.

Craniofacial photographic superimposition involves comparing a facial photograph of a missing person with a recovered skull. This technique is used primarily to exclude when photographs are available of a missing person thought perhaps to be represented by the recovered remains.

Recent research has focused on enhanced use of computers and related technology, as well as targeted efforts to clarify the relationship between soft and hard tissues. Facial approximation continues to represent a blend of art and science; however, recent advances have strengthened the scientific foundation.

Articles in this Special Issue of *Forensic Sciences Research* focus on overviews of the published literature on these topics. They also share results from the latest innovative research on these key areas of forensic anthropology applications.
